# Comparison of the Partition Efficiencies of Multiple Phenolic Compounds Contained in Propolis in Different Modes of Acetonitrile–Water-Based Homogenous Liquid–Liquid Extraction

**DOI:** 10.3390/molecules24030442

**Published:** 2019-01-26

**Authors:** Wenbin Chen, Xijuan Tu, Dehui Wu, Zhaosheng Gao, Siyuan Wu, Shaokang Huang

**Affiliations:** 1 College of Bee Science, Fujian Agriculture and Forestry University, Fuzhou 350002, China; xjtu@fafu.edu.cn (X.T.); dehui2580@163.com (D.W.); gzs100@126.com (Z.G.); wusiyuan2018@126.com (S.W.); skhuang@fafu.edu.cn (S.H.); 2 MOE Engineering Research Center of Bee Products Processing and Application, Fujian Agriculture and Forestry University, Fuzhou 350002, China

**Keywords:** salting-out assisted liquid–liquid extraction, sugaring-out assisted liquid–liquid extraction, hydrophobic-solvent assisted liquid–liquid extraction, subzero-temperature assisted liquid–liquid extraction, phenolic compounds

## Abstract

Homogeneous liquid–liquid extraction (HLLE) has attracted considerable interest in the sample preparation of multi-analyte analysis. In this study, HLLEs of multiple phenolic compounds in propolis, a polyphenol-enriched resinous substance collected by honeybees, were performed for improving the understanding of the differences in partition efficiencies in four acetonitrile–water-based HLLE methods, including salting-out assisted liquid–liquid extraction (SALLE), sugaring-out assisted liquid–liquid extraction (SULLE), hydrophobic-solvent assisted liquid–liquid extraction (HSLLE), and subzero-temperature assisted liquid–liquid extraction (STLLE). Phenolic compounds were separated in reversed-phase HPLC, and the partition efficiencies in different experimental conditions were evaluated. Results showed that less-polar phenolic compounds (kaempferol and caffeic acid phenethyl ester) were highly efficiently partitioned into the upper acetonitrile (ACN) phase in all four HLLE methods. For more-polar phenolic compounds (caffeic acid, *p*-coumaric acid, isoferulic acid, dimethoxycinnamic acid, and cinnamic acid), increasing the concentration of ACN in the ACN–H_2_O mixture could dramatically improve the partition efficiency. Moreover, results indicated that NaCl-based SALLE, HSLLE, and STLLE with ACN concentrations of 50:50 (ACN:H_2_O, *v*/*v*) could be used for the selective extraction of low-polarity phenolic compounds. MgSO_4_-based SALLE in the 50:50 ACN–H_2_O mixture (ACN:H_2_O, *v*/*v*) and the NaCl-based SALLE, SULLE, and STLLE with ACN concentrations of 70:30 (ACN:H_2_O, *v*/*v*) could be used as general extraction methods for multiple phenolic compounds.

## 1. Introduction

Increasing demands on monitoring a large number of target compounds have promoted the development of multi-analyte analytical methods. For instance, to assess a broad spectrum of possible metabolites, metabolomics requires multi-analyte methods to analyze the entire metabolome [1,2]. As another example, improper usage and the cross-contamination of chemicals in agricultural practice may lead to multi-residues of contaminants in agricultural products. Thus, multi-analyte methods have been developed to monitor the unknown chemical treatment history and protect the health of consumers [3]. Additionally, fingerprint profiles based on multi-analyte analysis of phytochemical compounds or volatile fractions in foods have been applied in foodomics for the issue of food quality [4].

Modern analytical instruments, especially the chromatography tandem mass spectrometry techniques, are capable of analyzing a large number of target compounds in a single analysis [5]. However, sample preparation procedure is still the crucial variable of multi-analyte analysis in achieving complete and accurate information [6]. Conventional liquid–liquid extraction has been widely used for the extraction of multiple low-polar compounds from aqueous sample solutions. Nevertheless, its applications in multi-analyte analysis are limited by the low extraction efficiency towards high-polarity compounds. Recently, homogeneous liquid–liquid extraction (HLLE) methods have been developed and extensively applied in multi-analyte analysis, due to their effective extraction of target compounds with a wide range of polarities [7,8,9]. In addition, HLLEs are receiving increasing interest from researchers because of the reduction of reagent consumption, extraction time, and the cost of analysis [7].

In acetonitrile–water-based HLLE, the acetonitrile (ACN) is mixed with water to form a homogenous solution for the extraction. Then, the ACN phase is triggered to partition from the aqueous solution with the addition of phase separation agents such as salts [8], sugars [10], hydrophobic solvents [11], or the cooling performance [12]. For example, Valente et al. reported the capabilities of salting-out assisted liquid–liquid extraction (SALLE) in phytochemical analysis [13]. This HLLE technique was demonstrated to be simple, of low cost, and versatile for the identification of various volatile and non-volatile compounds in fennel seeds (*Foeniculum vulgare Mill*). In addition, sugars can also trigger the phase separation in ACN–water mixtures, to develop the sugaring-out assisted liquid–liquid extraction (SULLE) method. Compared with SALLE, SULLE showed the advantage of being environment friendly [14]. Multi-analyte analysis of drugs in honey and plasma by using SULLE have been reported [15,16]. Recently, Liu et al. reported a similar phase separation phenomenon of ACN–water mixtures, induced by hydrophobic solvents [11]. This hydrophobic-solvent assisted liquid–liquid extraction (HSLLE) has been used for the profiling of endogenous phytohormones in plants [17]. Additionally, Yoshida et al. reported that ACN was separated from the aqueous solution at a subzero temperature (−20 °C) [12]. This technique, subzero-temperature assisted liquid–liquid extraction (STLLE), can avoid the residues of phase separation agents in the ACN phase, compared with the other three HLLE methods. This simple HLLE method has been applied for the determination of anthraquinone derivatives in sticky traditional Chinese medicines [18]. More recently, microextraction methods based on HLLE have been developed for the extraction and preconcentration of multiple contaminants in foodstuffs [19,20].

In multi-analyte analysis, understanding the distribution of compounds in the extractive is valuable for the design of sample preparation protocol to achieve a wide extraction of multi-analytes and minimize the co-extraction of interferences. Phenolic compounds play an important role in human diets, resulting from their nutritional significance and potentially beneficial health effects [21]. Despite HLLE having been widely used in multi-analyte analysis, little is known about the partition efficiencies of phenolic compounds in different acetonitrile–water-based HLLE methods, which are fundamentally important for the design of sample preparation protocol for analytical purposes. Propolis, a resinous substance collected by honeybees, is rich in polyphenols [22,23]. The reported methods for the extraction of phenolic compounds in propolis include maceration extraction [24], ultrasonic-assisted extraction [25], and microwave-assisted extraction [26]. In the present work, propolis is used as a model to systematically compare the partition efficiencies of phenolic compounds in four typical acetonitrile–water-based HLLE methods and discuss more details between the partition efficiencies and polarities of target phenolic compounds. To the best of our knowledge, this is the first report on the investigation of HLLE in propolis.

## 2. Results and Discussion

### 2.1. Salting-Out Assisted Liquid–Liquid Extraction

**Figure 1:**
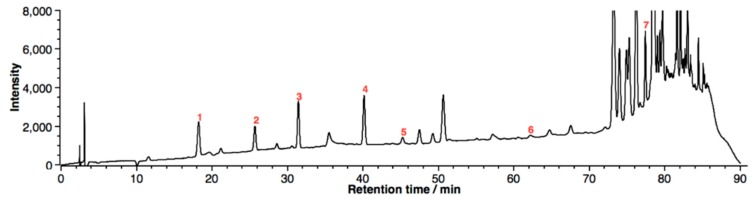
Representative HPLC-DAD chromatogram (λ = 280 nm) of phenolic compounds in propolis. 1, caffeic acid; 2, *p*-coumaric acid; 3, isoferulic acid; 4, dimethoxycinnamic acid; 5, cinnamic acid; 6, kaempferol; 7, caffeic acid phenethyl ester.

**Figure 2:**
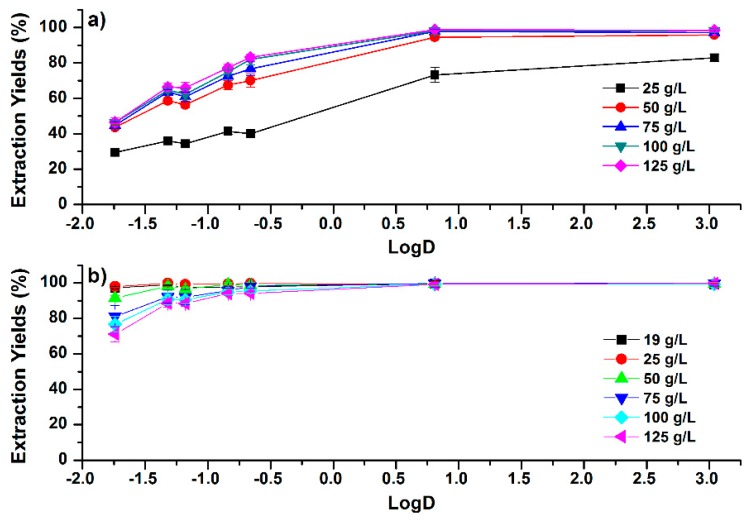
Correlations between extraction yields (EYs) and the LogD (pH 7.4) values of phenolic compounds under different addition amounts of (**a**) NaCl and (**b**) MgSO_4_ in acetonitrile–water (ACN–H_2_O) mixture (50:50, *v*/*v*). Error bars present the standard deviation (*n* = 3).

Seven typical phenolic compounds observed in propolis, including caffeic acid, *p*-coumaric acid, isoferulic acid, dimethoxycinnamic acid, cinnamic acid, kaempferol, and caffeic acid phenethyl ester (CAPE), were well-separated in reversed-phase HPLC, as shown in Figure 1. The chromatogram of standards is shown in Figure S1. Chromatographic peaks were identified based on the UV absorption spectra and the retention time compared to the standards. Effects of salt concentration on the partitioning of the phenolic compounds in the ACN–H_2_O mixture (50:50, *v*/*v*) are shown in Figure 2. To illustrate the correlation between partition efficiency and polarity, extraction yields (EYs) in the upper phase are plotted against the LogD (distribution coefficient) value of the estimated compounds, which were collected from ChemSpider [27] and shown in Table 1. Since the extraction solution was in the neutral pH, the value of LogD in pH 7.4 was selected. For NaCl-based SALLE, shown in Figure 2a, EYs of phenolic compounds increased as the salt concentration increased from 25 to 125 g/L. The detailed trends are shown in Figure S2a. When the concentration of NaCl was 25 g/L, EYs of the phenolic compounds were between 29.5% and 82.8%. Then, the EYs were dramatically raised to the range between 43.6% and 95.8% under the NaCl concentration of 50 g/L. As the salt concentration further increased to 125 g/L, slight growth in EYs was observed. Additionally, trends of increasing EYs with the increase of LogD values were observed at each salt concentration. The maximum EYs were found in the least polar compound (CAPE), while the minimum EYs were observed in the most polar compound (caffeic acid).

Compared with NaCl, lower salt concentrations of MgSO_4_ are required to trigger the phase separation. In addition, higher EYs are achieved for all the estimated phenolic compounds in MgSO_4_-based SALLE. As shown in Figure 2b, EYs of the phenolic compounds were all larger than 97% when the concentration of MgSO_4_ was 19 g/L in ACN–H_2_O mixture (50:50, *v*/*v*). This higher partition efficiency of MgSO_4_ compared with NaCl was similar with reports on the SALLE of dicarbonyl [28] and fatty acid [29] compounds, which can be attributed to the larger phase ratio in MgSO_4_-based SALLE than that in NaCl-based SALLE. As shown in Figure 2, EYs of more-polar phenolic compounds with LogD < 0 (caffeic acid, *p*-coumaric acid, isoferulic acid, dimethoxycinnamic acid, and cinnamic acid) in NaCl-based SALLE are lower than those in MgSO_4_. However, EYs of the less-polar phenolic compounds with logD > 0 (kaempferol and CAPE) in NaCl-based SALLE are similar with those in MgSO_4_-based SALLE. This means that though the phase ratio is lower in NaCl-based SALLE [28], EYs of less-polar compounds are comparable to the high phase ratio conditions of MgSO_4_-based SALLE. Additionally, when the concentration of MgSO_4_ was increased to 125 g/L, the volume of the upper phase was decreased. This decrease of phase ratio with the increase in MgSO_4_ concentration is consistent with the results reported by Valente et al. [28]. Consequently, the decrease in the EY of the most polar compound (caffeic acid) was from 98.0% to 71.2%, while for the least polar compound (CAPE), EY varied between 99.3% and 99.8% (Figure S2b). These observations indicate that the influence of the phase ratio on partition efficiency is more significant when the polarity of the estimated compounds is higher. It also implies that EYs of polar compounds may be improved by increasing the phase ratio.

**Figure 3:**
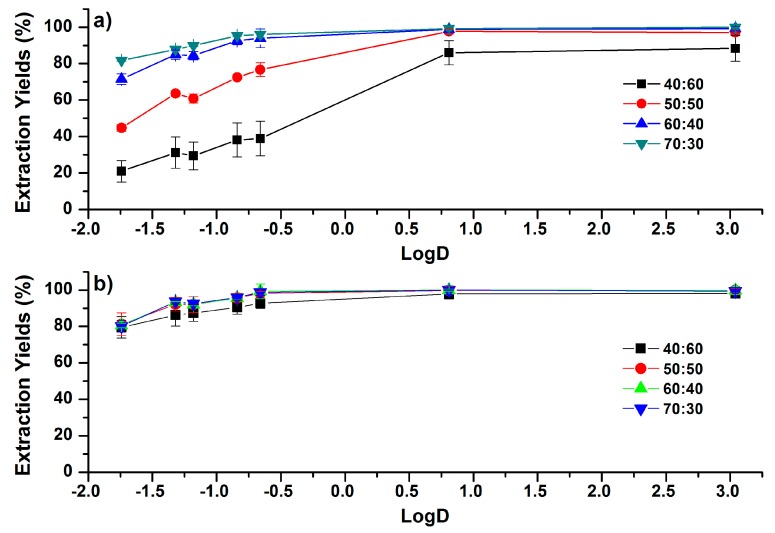
Correlations between EYs and the LogD (pH 7.4) values of phenolic compounds under different concentration of acetonitrile (ACN) in the ACN–H_2_O mixture (ACN:H_2_O, *v*/*v*) for (**a**) NaCl- and (**b**) MgSO_4_-based salting-out assisted liquid–liquid extraction (SALLE). The addition amounts of salts were 75 g/L. Error bars present the standard deviation (*n* = 3).

Increasing the initial concentration of ACN in ACN–H_2_O mixtures can lead to the increase of phase ratio [29]. Consequently, the improvement of EYs for more-polar compounds were observed. For NaCl-based SALLE, as shown in Figure 3a, when the ACN concentration in the ACN–H_2_O mixture was increased to 70:30 (ACN:H_2_O, *v*/*v*), the EYs of phenolic compounds were all higher than 81.8%. In addition, the influence of initial ACN concentration on EYs is more significant for more-polar phenolic compounds than for less-polar phenolic compounds. For instance, as the initial concentration of ACN increased from 40:60 to 70:30 (ACN:H_2_O, *v*/*v*), EYs of caffeic acid were increased from 21.0% to 81.8%, while for CAPE, EYs were increased from 88.4% to 99.9%. In MgSO_4_-based SALLE, as shown in Figure 3b, the influence of the initial concentration of ACN on EYs is much lower than that in NaCl-based SALLE. As the concentration of ACN increased from 40:60 to 70:30, EYs of caffeic acid and CAPE in MgSO_4_-based SALLE varied from 79.5% to 81.2% and from 98.1% to 99.8%, respectively.

From these above experiments, it becomes clear that MgSO_4_-based SALLE with low salt concentration may be used as a general HLLE method for compounds with a wide range of polarities because of the high EYs achieved (>98%) for all the investigated compounds with LogD ≥ −1.74. NaCl-based SALLE with an ACN concentration of 70:30 in the ACN–H_2_O mixture (ACN:H_2_O, *v*/*v*) may also be a general extraction method, as the EYs were not less than 90% for investigated compounds with LogD ≥ −1.18. If the multi-analyte compounds are less polar, NaCl-based SALLE with a salt concentration of 50 g/L and an ACN concentration of 50:50 would be a suitable choice. In this condition, EYs were larger than 94% for the investigated compounds with LogD ≥ 0.81. These EYs values are comparable with MgSO_4_-based SALLE. Furthermore, the volume of the upper phase is lower than that in MgSO_4_-based SALLE. This could be helpful for enhancing the sensitivity of analysis, and the lower EYs towards polar compounds in this condition would reduce the co-extraction of high-polarity interferences.

### 2.2. Sugaring-Out Assisted Liquid–Liquid Extraction

**Figure 4:**
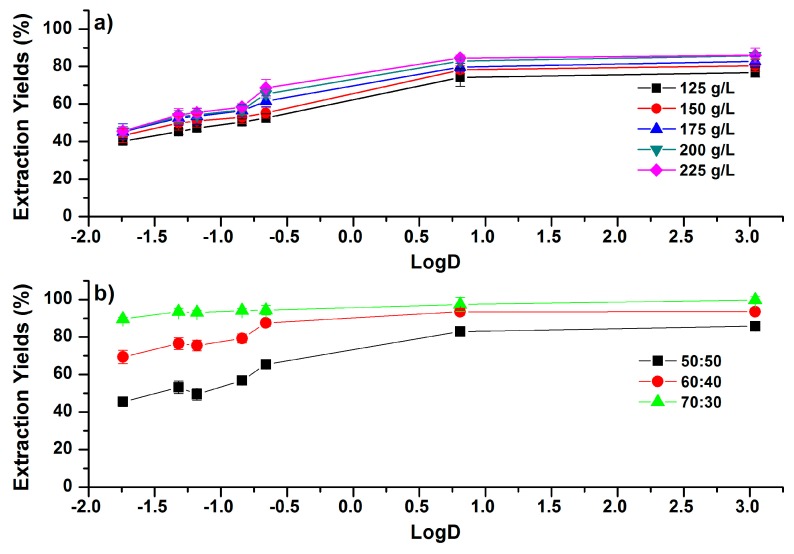
Correlations between EYs and the LogD (pH 7.4) values of phenolic compounds (**a**) under different addition amounts of glucose in ACN–H_2_O mixtures (50:50, *v*/*v*); (**b**) under different concentrations of ACN in the ACN–H_2_O mixture (ACN:H_2_O, *v*/*v*) with the addition of 200 g/L glucose. Error bars present the standard deviation (*n* = 3).

Sugars, including glucose, fructose, and sucrose etc., have been reported to trigger the phase separation of ACN–H_2_O mixtures [14]. Glucose was chosen in this study because it has been demonstrated to be the better phase separation agent than other sugars [10]. Effects of glucose concentration on the partition efficiencies of phenolic compounds in ACN–H_2_O mixtures (50:50, *v*/*v*) are shown in Figure 4a. Results indicated that EYs were increased as more glucose was introduced. The required amount of glucose to trigger the phase separation is larger than the required amount of NaCl and MgSO_4_. Increasing the concentration of glucose from 125 to 225 g/L led to the increase of EYs, and the obtained maximum values of EYs ranged from 45.6% (caffeic acid) to 86.1% (CAPE). The detailed trends are shown in Figure S3. In addition, EYs of the phenolic compounds displayed the trends of increasing as the value of LogD increased at each glucose concentration, which are similar with the trends in NaCl-based SALLE.

Effects of the initial concentration of ACN on EYs are shown in Figure 4b. Increasing the concentration of ACN in ACN–H_2_O mixtures (ACN:H_2_O, *v*/*v*) from 50:50 to 70:30 significantly increased the EYs of phenolic compounds, and the increment appeared to decrease as the polarity of the compounds became less polar. For instance, EYs of the most polar compound (cafferic acid) were increased from 45.5% to 89.6%, while EYs of the least polar compound (CAPE) were increased from 85.8% to 99.7%. When the concentration of ACN was 70:30 (ACN:H_2_O, *v*/*v*), EYs were in the range of 90% (cafferic acid) to 99.7% (CAPE). The obtained EYs for more-polar compounds in SULLE are higher than NaCl-based SALLE, and are comparable with MgSO_4_-based SALLE. This could be attributed to the high phase ratio under the condition of high ACN concentration in SULLE [29].

It is important to note that SULLE may work as a general method under the ACN concentration of 70:30 (ACN:H_2_O, *v*/*v*) with a glucose concentration of 200 g/L. In this condition, investigated phenolic compounds with LogD ≥ −1.74 could be partitioned into the upper phase, with EYs not less than 90%. SULLE in the high ACN concentration may be used as an alternative method for MgSO_4_-based SALLE, as the volume of the upper phase and the obtained EYs are comparable with MgSO_4_.

### 2.3. Hydrophobic-Solvent Assisted Liquid–Liquid Extraction

**Figure 5:**
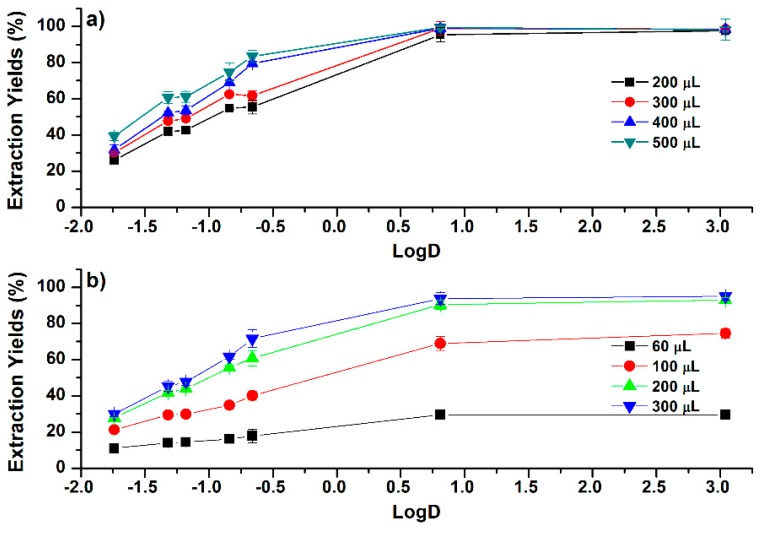
Correlations between EYs and the LogD (pH 7.4) values of phenolic compounds under different addition volumes of (**a**) dichloromethane (DCM) and (**b**) chloroform in ACN–H_2_O mixtures (50:50, *v*/*v*). Error bars present the standard deviation (*n* = 3).

In HSLLE, two typical hydrophobic solvents, dichloromethane (DCM) and chloroform, were studied. The volume of DCM required to trigger the phase separation is larger than that of chloroform. The investigated volumes for the HSLLE were in the range of 200 to 500 µL and 60 to 300 µL for DCM and chloroform, respectively. As shown in Figure 5a, EYs of the more-polar phenolic compounds were increased with the introduction of more DCM into the ACN–H_2_O mixture (50:50, *v*/*v*), whereas EYs of the less-polar phenolic compounds were slightly varied, with values larger than 92%. Furthermore, the influence of solvent volume on EYs was more significant for chloroform than for DCM. As the volume of chloroform increased from 60 to 300 µL, EYs of cafferic acid were increased from 11.0% to 29.9%, and EYs of CAPE were increased from 29.6% to 95.0%, as shown in Figure 5b. This observation might be attributed to the significant increase of the upper phase volume when more chloroform is introduced [11]. It is interesting to find that EYs of more-polar phenolic compounds are much lower than those of less-polar phenolic compounds under the introduction of DCM or chloroform. This means that HSLLE may be used for the selective extraction of less-polar phenolic compounds.

**Figure 6:**
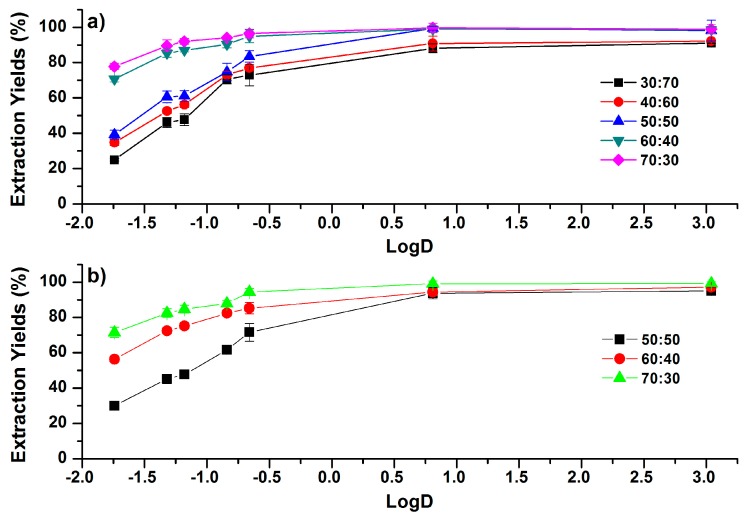
Correlations between EYs and the LogD (pH 7.4) values of phenolic compounds under different concentration of ACN in the ACN–H_2_O mixture (ACN:H_2_O, *v*/*v*) for (**a**) DCM and (**b**) chloroform. The addition volumes of DCM and chloroform were 25% and 15% of the initial volume of ACN in the ACN–H_2_O mixture, respectively. Error bars present the standard deviation (*n* = 3).

Increasing the initial concentration of ACN results in the increase of EYs in both DCM and chloroform HSLLEs, as shown in Figure 6. The increments of EYs were dramatic for more-polar phenolic compounds, but the increment reduced as the polarity of the compounds decreased. As the concentration of ACN (ACN:H_2_O, *v*/*v*) increased from 30:70 to 70:30 in DCM-based HSLLE, as shown in Figure 6a, EYs of caffeic acid and CAPE increased from 25.0% to 77.8% and from 91.0% to 99.0%, respectively. In chloroform-based HSLLE, shown in Figure 6b, EYs of caffeic acid and CAPE increased from 30.0% to 71.4% and 95.0% to 99.2%, respectively, when the concentration of ACN (ACN:H_2_O, *v*/*v*) increased from 50:50 to 70:30. Therefore, EYs of the more-polar phenolic compounds display more sensitivity to the increase of the ACN concentration than less-polar phenolic compounds, which has also been found in the above results of SALLE and SULLE.

Compared with SULLE and SALLE, HSLLE shows the better selective extraction of less-polar phenolic compounds. With the addition of 200 µL DCM and chloroform, EYs of the investigated compounds with LogD > 0.81 in the ACN–H_2_O mixture (50:50, *v*/*v*) were higher than 95% and 90%, respectively. Furthermore, EYs of polar compounds are much lower, and thus the co-extraction of high-polarity interference compounds might be dramatically reduced.

### 2.4. Subzero-Temperature Assisted Liquid–Liquid Extraction

**Figure 7:**
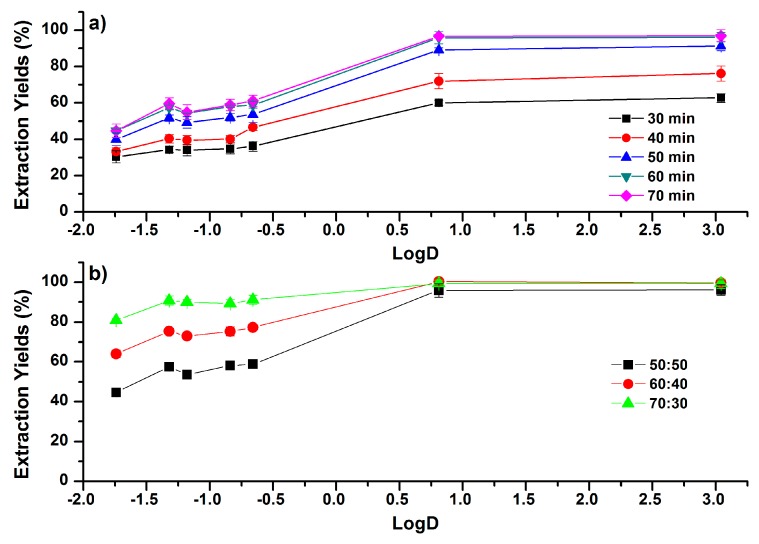
Correlations between EYs and the LogD (pH 7.4) values of phenolic compounds (**a**) under different cooling times in ACN–H_2_O mixture (50:50, *v*/*v*); (**b**) under different concentrations of ACN in the ACN–H_2_O mixture (ACN:H_2_O, *v*/*v*) with a cooling time of 60 min. Error bars present the standard deviation (*n* = 3).

In STLLE, the extraction solution of ACN–H_2_O mixture was cooled at a low temperature (−20 °C) to induce the phase separation. The cooling time significantly influences the partition performance [12]. As shown in Figure 7a, EYs of the phenolic compounds increased with the extending of cooling time. The phase separation began at the cooling time of 30 min. The volume of the upper phase was increased as the cooling time extended, and the lower aqueous phase was nearly frozen at 60 min. Consequently, EYs were increased with the extending of cooling time, and reached the plateau at 60 min. In addition, increments of EYs in less-polar phenolic compounds were larger than those of more-polar phenolic compounds. For example, EYs of cafferic acid and CAPE were increased from 30.4% to 44.8% and 62.8% to 97.0%, respectively. Moreover, STLLE showed the selective partitioning of less-polar phenolic compounds. When the phase separation was performed in the ACN–H_2_O mixture (50:50, *v*/*v*) under 60 min cooling, EYs of more-polar compounds were under 61%, while the EYs of investigated compounds with LogD ≥ 0.81 were higher than 96%.

Increasing the initial concentration of ACN in the ACN–H_2_O mixture also results in the increase of EYs in STLLE, as shown in Figure 7b. The influences of ACN concentration on EYs are more significant in more-polar phenolic compounds than in less-polar phenolic compounds. As the concentration of ACN increased from 50:50 to 70:30 (ACN:H_2_O, *v*/*v*), EYs of caffeic acid and CAPE increased from 44.7% to 80.8% and from 96.1% to 99.3%, respectively. Additionally, EYs of the investigated compounds were all increased to be larger than 80.8% when the initial concentration of ACN was 70:30 (ACN:H_2_O, *v*/*v*). This observation implies that STLLE, at high initial concentrations of ACN, may be applied for the extraction of wide-polarity multi-analyte compounds with high EYs. The drawback of STLLE might be the relatively long time for the complete phase separation, but it shows the advantages of having a simple procedure, easy collection of the upper phase, and the elimination of additional phase separation agents.

## 3. Materials and Methods

### 3.1. Materials

Methanol and ACN with HPLC grades were obtained from Merck (Darmstadt, Germany). Standards of caffeic acid, *p*-coumaric acid, isoferulic acid, dimethoxycinnamic acid, cinnamic acid, kaempferol, and caffeic acid phenethyl ester (CAPE) were purchased from Aladdin (Shanghai, China). Anhydrous magnesium sulfate, sodium chloride, glucose, anhydrous ethanol, dichloromethane (DCM), and chloroform were all of analytical grade and obtained from Sinopharm Chemical Reagent Co., Ltd (Shanghai, China). Ultrapure water (18.2 MΩ) was used throughout this article. Raw propolis was collected from Hubei, China.

### 3.2. Homogeneous Liquid–Liquid Extraction

Raw propolis was purified according to the reported method to remove the insoluble subjects and wax compounds [30]. Raw propolis was frozen and ground prior to the extraction. The ground samples were extracted by maceration for 7 days at room temperature, with 10 mL anhydrous ethanol for every 3 g of raw propolis. The insoluble subjects were separated by filtration. The filtrates were frozen overnight and filtered again to remove the wax compounds. Solvent was then evaporated on a water bath at 50 °C to obtain dry extracts of propolis. Purified propolis (100 mg) was weighted into the 100 mL volumetric flask and diluted to the volume with different ACN–water mixtures, and sonicated for 20 min. For SALLE, SULLE, and HSLLE, this propolis solution (4 mL) was added with different amounts of phase separation agents (NaCl and MgSO_4_ for SALLE, glucose for SULLE, and DCM and chloroform for HSLLE) and then vortexed for 1 min. The mixed solution was centrifuged at 6000 rpm for 5 min to make a clear phase separation. The upper phase was collected by a micro-syringe and then diluted to 25 mL with anhydrous ethanol. For STLLE, the propolis solution (4 mL) was cooled at −20 °C for different times. The upper phase was repeatedly collected and transferred into a 25 mL volumetric flask by micro-syringe (100 μL), and then diluted to 25 mL with anhydrous ethanol. This dilution procedure was performed to make all the collected upper phases into the same volume. These final extractive solutions were analyzed by HPLC. Another propolis solution (4 mL) without phase separation was diluted to 25 mL with anhydrous ethanol. This control solution was also analyzed by HPLC and used for the calculation of extraction yields. All experiments were triplicates.

### 3.3. HPLC Analysis

Phenolic compounds were analyzed based on the reversed-phase HPLC method reported by Zhang et al. [31]. The HPLC system (Shimadzu, Kyoto, Japan) was composed of LC-20AT pumps, a SIL-20AC autosampler, a CTO-20AC column oven, and a SPD-M20A photodiode array detector (190~800 nm). A Wonda Cract (Shimadzu-GL) C18 column (5 μm, 4.6 × 150 mm) was used for the separation. The mobile phase A was 0.1% aqueous acetic acid solution (*v*/*v*) and the mobile phase B was methanol. Gradient elution was as follows: 15–40% B at 0–30 min, 40–55% B at 30–65 min, 55–62% B at 65–70 min, 62–100% B at 70–80 min, 100–15% at 80–85 min, and stayed at 15% for 5 min. The flow rate was 0.8 mL/min, the injection volume was 10 μL, and the column temperature was 35 °C. Standards were used for the identification of chromatographic peaks. The partition efficiency was compared by extraction yields (EYs, %) = (peak areas of target compounds in the HLLE extractive solution/peak areas of target compounds in the control solution) × 100. A chromatogram at 280 nm was used for the calculation of peak area.

## 4. Conclusions

In summary, we present the first report of HLLE in propolis. Partitioning of seven phenolic compounds in the ACN–H_2_O mixture, triggered by four HLLE methods, were investigated. The partition efficiencies were found to be correlated with the polarity of the target compounds. The less-polar phenolic compounds (kaempferol and caffeic acid phenethyl ester) could be highly efficiently partitioned into the upper ACN phase in all four investigated HLLE methods. For more-polar phenolic compounds (caffeic acid, *p*-coumaric acid, isoferulic acid, dimethoxycinnamic acid, and cinnamic acid), increasing the initial concentration of ACN in the ACN–H_2_O mixture is suggested for archiving higher EYs. This study has also suggested that MgSO_4_-based SALLE under the ACN concentration of 50:50 (ACN:H_2_O, *v*/*v*), together with NaCl-based SALLE, SULLE, and STLLE under the ACN concentration of 70:30 (ACN:H_2_O, *v*/*v*) might be used as general HLLE methods for the extraction of multiple phenolic compounds with a wide range of polarities. Additionally, NaCl-based SALLE, HSLLE, and STLLE with an ACN concentration of 50:50 (ACN:H_2_O, *v*/*v*) might be used for the selective extraction of low-polarity phenolic compounds. These observations show a better understanding of the partitioning of multiple phenolic compounds in HLLE methods, and would be valuable for the development of sample preparation protocol in phytoanalysis.

## Figures and Tables

**Table 1:** LogD (pH 7.4) values of the phenolic compounds.

Compounds	LogD (pH 7.4) ^a^
Caffeic acid	−1.74
*p*-Coumaric acid	−1.32
Isoferulic acid	−1.18
Dimethoxycinnamic acid	−0.84
Cinnamic acid	−0.66
Kaempferol	0.81
Caffeic acid phenethyl ester	3.04

^a^ Data were collected from [27].

## Supplementary Materials

The following are available online: Figure S1: Representative HPLC-DAD chromatogram (λ = 280 nm) of phenolic standards. 1, caffeic acid; 2, *p*-coumaric acid; 3, isoferulic acid; 4, dimethoxycinnamic acid; 5, cinnamic acid; 6, kaempferol; 7, caffeic acid phenethyl ester. Figure S2: Extraction yields of investigated phenolic compounds under different addition amounts of (**a**) NaCl and (**b**) MgSO_4_ in ACN–H_2_O mixture (50:50, *v*/*v*). Error bars present the standard deviation (*n* = 3). Figure S3: Extraction yields of investigated phenolic compounds under different addition amounts of glucose in ACN–H_2_O mixture (50:50, *v*/*v*). Error bars present the standard deviation (*n* = 3).
