# Supplementary material for: Comparison of the Partition Efficiencies of Multiple Phenolic Compounds Contained in Propolis in Different Modes of Acetonitrile–Water-Based Homogenous Liquid–Liquid Extraction
Source: Molecules. 2019 Jan 26;24(3):442. doi: 10.3390/molecules24030442 (PMC6384799; doi:10.3390/molecules24030442)
Supplement: Supplementary file 1 [file molecules-24-00442-s001.pdf]

molecules

*Supplementary material*

# Comparison of the partition efficiency of multiple phenolic compounds contained in propolis in different modes of acetonitrile-water based homogenous liquid-liquid extraction

**Wenbin Chen [1,2,*], Xijuan Tu [1,2], Dehui Wu [1], Zhaosheng Gao [1], Siyuan Wu [1], and Shaokang Huang [1]**

[1] College of Bee Science, Fujian Agriculture and Forestry University, Fuzhou, China.
[2] MOE Engineering Research Center of Bee Products Processing and Application, Fujian Agriculture and Forestry University, Fuzhou, China.
* Correspondence: wbchen@fafu.edu.cn; Tel.: +86-591-83789482

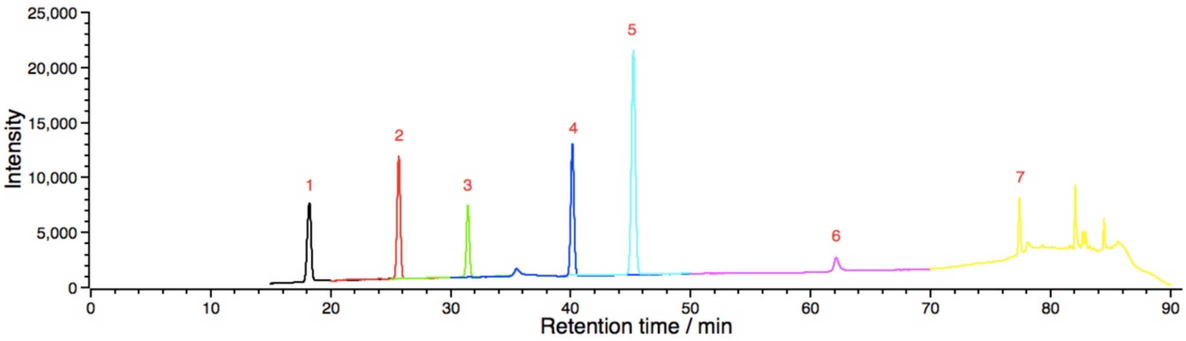


**Figure S1.** Representative HPLC-DAD chromatogram ($\lambda$=280 nm) of phenolic standards. 1, caffeic acid; 2, *p*-coumaric acid; 3, isoferulic acid; 4, dimethoxycinnamic acid; 5, cinnamic acid; 6, kaempferol; 7, caffeic acid phenethyl ester.

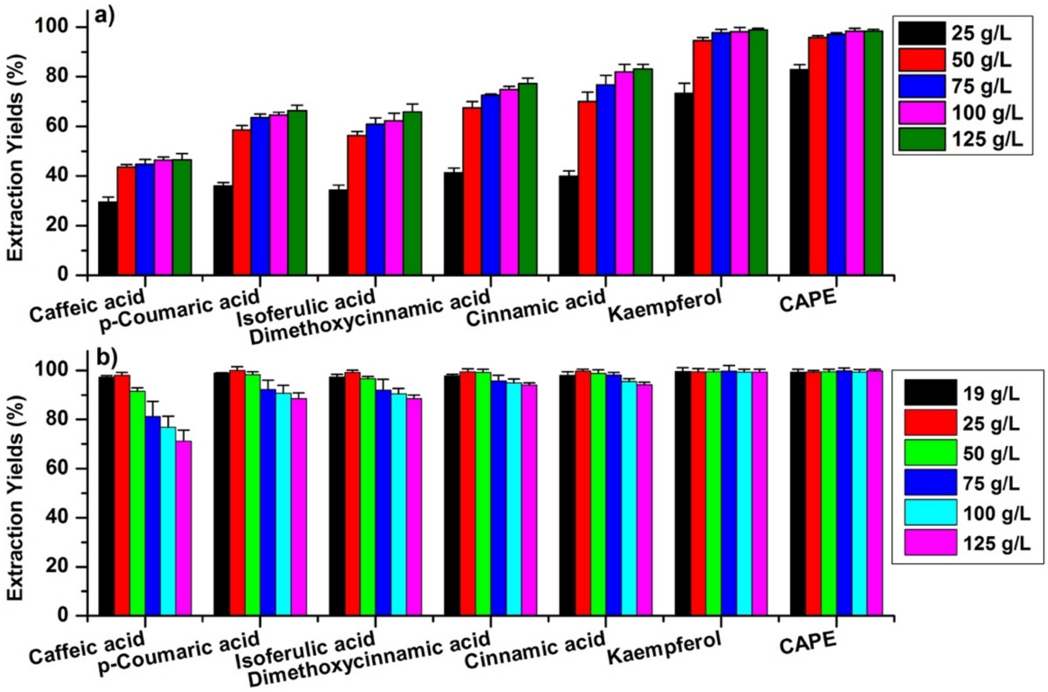


**Figure S2.** Extraction yields of investigated phenolic compounds under different addition amounts of a) NaCl and b) $MgSO_4$ in ACN-$H_2O$ mixture (50:50, v/v). Error bars present the standard deviation (n=3).

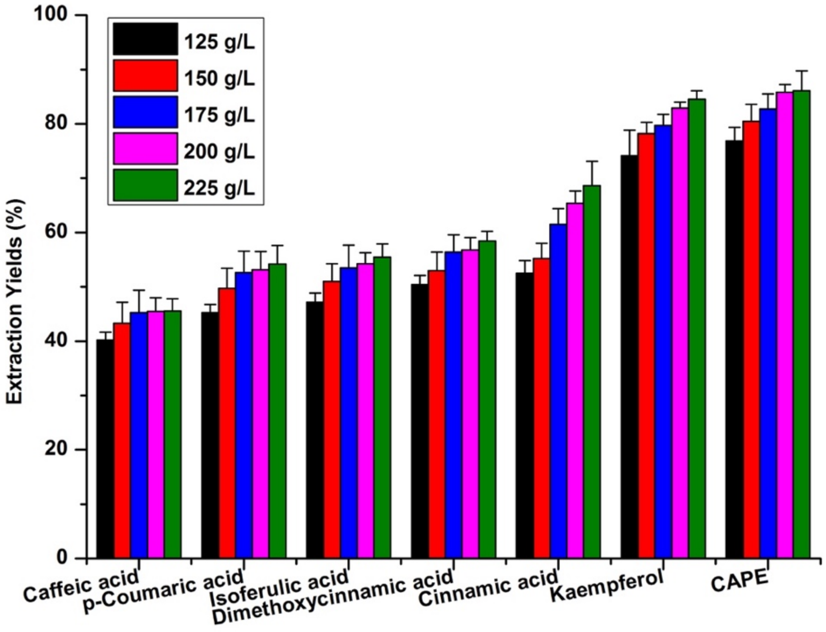


**Figure S3.** Extraction yields of investigated phenolic compounds under different addition amounts of glucose in ACN-$H_2O$ mixture (50:50, v/v). Error bars present the standard deviation (n=3).
